# A single-base mutation in promoter of *CsTPR* enhances the negative regulation on mechanical-related leaf drooping in tea plants

**DOI:** 10.1093/hr/uhaf098

**Published:** 2025-03-25

**Authors:** Haoran Liu, Lingxiao Duan, Chaqin Tang, Jianqiang Ma, Ji-Qiang Jin, Jiedan Chen, Weizhong He, Mingzhe Yao, Liang Chen

**Affiliations:** Key Laboratory of Biology, Genetics and Breeding of Special Economic Animals and Plants, Ministry of Agriculture and Rural Affairs, Tea Research Institute of the Chinese Academy of Agricultural Sciences, No. 9, Meiling South Road, Hangzhou, Zhejiang 310008, China; Yunnan Key Laboratory of Tea Germplasm Conservation and Utilization in the Lancang River Basin, West Yunnan University, No. 2, Xuefu Road, Lincang, Yunnan 677000, China; Key Laboratory of Biology, Genetics and Breeding of Special Economic Animals and Plants, Ministry of Agriculture and Rural Affairs, Tea Research Institute of the Chinese Academy of Agricultural Sciences, No. 9, Meiling South Road, Hangzhou, Zhejiang 310008, China; Jiangsu Vocational College of Agriculture and Forestry, No. 19, Wenchang East Road, Jurong, Jiangsu 212400, China; Key Laboratory of Biology, Genetics and Breeding of Special Economic Animals and Plants, Ministry of Agriculture and Rural Affairs, Tea Research Institute of the Chinese Academy of Agricultural Sciences, No. 9, Meiling South Road, Hangzhou, Zhejiang 310008, China; Key Laboratory of Biology, Genetics and Breeding of Special Economic Animals and Plants, Ministry of Agriculture and Rural Affairs, Tea Research Institute of the Chinese Academy of Agricultural Sciences, No. 9, Meiling South Road, Hangzhou, Zhejiang 310008, China; Key Laboratory of Biology, Genetics and Breeding of Special Economic Animals and Plants, Ministry of Agriculture and Rural Affairs, Tea Research Institute of the Chinese Academy of Agricultural Sciences, No. 9, Meiling South Road, Hangzhou, Zhejiang 310008, China; Lishui Institute of Agriculture and Forestry Sciences, No. 827, Liyang Street, Lishui, Zhejiang 323000, China; Key Laboratory of Biology, Genetics and Breeding of Special Economic Animals and Plants, Ministry of Agriculture and Rural Affairs, Tea Research Institute of the Chinese Academy of Agricultural Sciences, No. 9, Meiling South Road, Hangzhou, Zhejiang 310008, China; Key Laboratory of Biology, Genetics and Breeding of Special Economic Animals and Plants, Ministry of Agriculture and Rural Affairs, Tea Research Institute of the Chinese Academy of Agricultural Sciences, No. 9, Meiling South Road, Hangzhou, Zhejiang 310008, China; Yunnan Key Laboratory of Tea Germplasm Conservation and Utilization in the Lancang River Basin, West Yunnan University, No. 2, Xuefu Road, Lincang, Yunnan 677000, China

## Abstract

Mechanical harvesting in the tea industry has become increasingly essential due to its advantages in increasing productivity and reducing labor costs. Leaf droopiness caused a high rate of broken leaves, hindering the mechanized harvesting quality. However, the underlying mechanisms remain unclear. We herein identified a quantitative trait locus, designated as *q10.3*, along with three lead single nucleotide polymorphisms (SNPs) located near a *TPR* gene (*TETRATRICOPEPTIDE REPEAT*), named *CsTPR*, through performing a genome-wide association study (GWAS) on 130 tea accessions. Integrated analysis of RNA-seq and ATAC-seq confirmed *CsTPR* as a droopiness-associated candidate gene at the transcriptional level. *CsTPR* was then proved to negatively regulate brassinosteroid-induced droopiness by using the *CsTPR*-silencing tea plant. Whole-genome sequencing (WGS) combined with genome walking further indicated that a single-base mutation (T–A) in the promoter of *CsTPR*. ChIP-seq revealed that this mutation occurred within the binding site, E-box, of CsBES1.2 on the *CsTPR* promoter. Notably, CsBES1.2 bound the E-box of *CsTPR* promoter to repress the expression of *CsTPR*, as demonstrated by chromatin immunoprecipitation quantitative polymerase chain reaction (ChIP-qPCR), electrophoretic mobility shift assays (EMSA), and transient assays. The single-base mutation strengthened the inhibitory effect of CsBES1.2 on the expression of *CsTPR* via enhancing the binding affinity to the E-box. Altogether, our findings suggest that CsTPR negatively regulates droopiness in tea plants under the transcriptional repression of CsBES1.2 and that a single-base mutation within E-box amplifies the suppression of CsBES1.2 on the expression of *CsTPR*.

## Introduction

Tea, marketed as tender leaves harvested and processed from the tea plant (*Camellia sinensis* (L.) O. Kuntze), is one of the most popular beverages worldwide [1]. Harvesting represents the critical first-stage determinant of commercial tea quality [2], and the primary forms of harvesting can typically be divided into hand plucking and machine cutting [1,3,4]. Due to an acute labor shortage caused by an aging population, the mainstream method of tea production is shifting from labor-intensive hand plucking to technology-intensive mechanical harvesting.

Mechanized harvesting requires appropriate agronomy traits of tea plants, including longer internode length and straight leaves, to prevent leaf damage that can occur during machine operation [3,4]. Research on mechanical harvesting in various crops indicates that both appropriate machinery and suitable cultivars are equally important [5,6]. However, current tea plant cultivars were not developed with features suitable for mechanical harvesting since hand plucking was the traditional method used in the past. To breed cultivars that are suitable for mechanical harvesting, understanding the regulation network on harvesting-related traits was one of the foundations for directing breeding orientation. Past studies have indicated that phytohormones, such as brassinosteroid (BR) and gibberellin, were involved in the regulation of harvesting-related traits in tea plants [3,4]. For instance, BR has been shown to induce droopy leaves in tea plants by activating the signaling component CsBES1.2, which affects the expression of *CsEXL3* [3]. However, there is still limited information regarding the regulators of droopiness in tea plants, and more details on the related genes within these regulatory networks need to be uncovered.

*TPR* (*TETRATRICOPEPTIDE REPEAT*) protein contained a 34 amino acid motif that is loosely based around the consensus residues -W-LG-Y-A-F-A-P- [7]. TTLs (TETRATRICOPEPTIDE THIOREDOXIN-LIKE) were regarded as one special group of TPRs characterized by a CXXC active site at their N-terminal [8]. Hundreds of TPRs have been annotated in single plant species, such as rice and Arabidopsis. Some of these proteins are known to function in chloroplast development [9], cell wall remodeling in later root development [10], resistance to abiotic stress [11,12] and participant in phytohormone signal [13]. TPRs have a close relationship with various phytohormones, like ethylene, gibberellin, and BR, to control different traits in plants. For example, the TPR gene, ETO1, negatively regulates ethylene biosynthesis through interaction with both E3 ligase, AtCUL3, and ethylene synthetase, AtACS5 [14]. Additionally, AtTRP1 altered ethylene signaling as well as changed expression pattern of auxin-responsive genes [15]. Another TPR repeat-containing protein, SPINDLY, functioned as a negative regulator of GA signaling in *Arabidopsis* [16]. TTL3, one of four TTLs in *Arabidopsis*, interacted with BRI1 to promote BR responses [17].

The regulation of BRs on leaf droopiness in tea plants, along with the role of TPRs in the BR signaling pathway, highlights the need to further explore the function of TPRs in BR-induced droopiness. In this study, we conducted a conjoint analysis using genome-wide association studies (GWAS), RNA sequencing (RNA-seq), and assay for transposase-accessible chromatin using sequencing (ATAC-seq) to identify a droopiness-related quantitative trait locus (QTL) near the promoter of CsTPR in tea plants. The negative functions of CsTPR on droopiness were further determined by utilizing a *CsTPR-silencing* tea plant model. The relationship between the single-base mutation in the promoter of *CsTPR* and its expression was also established. Molecular interaction experiments were performed to show the negative transcriptional regulation of CsBES1.2 on the expression of *CsTPR*, illustrating the regulatory gap caused by the aforementioned single-base mutation. Our findings provide the first evidence that CsTPR depresses BR-induced leaf droopiness and the negative regulation of the BR signaling component on CsTPR. This research enhances our understanding of droopiness regulation in tea plant development, which is beneficial for improving mechanical harvesting practices.

## Results

### Identification of *CsTPR* as a candidate gene for droopy leaves through GWAS

**Figure 1:**
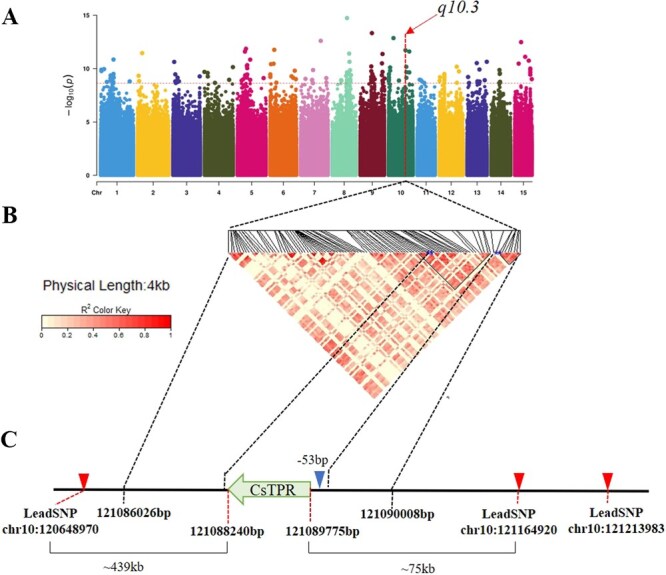
GWAS of droopy leaves. (A) Manhattan plot according to GWAS for PF. The red horizontal line indicated the threshold (−log_10_(*P*), *P*-value = 2.34E-09). The vertical line indicated the QTL, *q10.3*. *q10.3* included three lead SNPs, as shown in (C). (B) LD plot of 100 SNPs between lead SNPs in a 0.4-Mb region (121086026–121090008 bp) of chromosome 10. Asterisk indicated the nearest SNPs to the initiation (121089783/121089905 bp) and stop code (121088235/121088244 bp) of CsTPR. (C) Gene structure of CsTPR. The direction of arrow indicated the translation direction of CsTPR. The initiation site and stop site were 121089775 and 121088240 bp, respectively. Arrow indicated the location at −53 bp promoter of *CsTPR*. The detailed distance from the lead SNP to the gene was drawn under this figure.

Droopy leaves on tea plants obstruct the path of blade-cutting, which leads to an increase in the rate of broken leaves and reduces the efficiency of machine harvesting [3]. While some positive regulatory factors regulating curve blades have been identified [3], negative regulatory factors remain largely unknown. To unveil these factors, we employed 130 tea plant accessions with either straight or droopy leaves in NTGRH (the National Tea Germplasm Repository Hangzhou) for GWAS (Supplemental Table S1). PF values of all these genotyped accessions were documented in previous research [3]. EMMAX was more efficient to analyze large-scale data via using approximate methods to calculate random effects. The mixed model includes fixed effects and random effects to correct the correlation between samples. Using the genome-wide efficient mixed-model association (EMMAX) model, one of 16 QTLs, *q10.3*, with three lead single nucleotide polymorphisms (SNPs) (chr10:120648970, *P*-value = 6.78 × 10^−11^; chr10:121164920, *P*-value = 7.29 × 10^−10^; chr10:121213983, *P*-value = 2.14 [10] on chromosome 10 was selected (Fig. 1; Supplemental Fig. S3). Eleven genes were found between lead SNP chr10:120648970 and chr10:121213983. Among these genes, *CsTPR* (*TETRATRICOPEPTIDE REPEAT*) exhibited the highest value of FPKM in three leaf tissues, including the young leaf, mature leaf, and old leaf (Supplemental Table S2), according to the gene expression data of ‘Shuchazao’ obtained from TeaGVD [18]. Subsequent analysis of the pairwise linkage disequilibrium (LD) estimates (rs^2^) revealed the extent of LD decay within the region of 121088235/121088244 bp–121089783/121089905 bp (indicated by blue asterisks marking the starting and stopping positions of *CsTPR* coding sequence) as well as in the promoter region of CsTPR (Fig. 1B). Only one gene, *CsTPR*, was located in this region on the antisense strand (121089775–121088240 bp) without any intron (Fig. 1C). This evidence suggested CsTPR as a candidate gene for regulating droopy leaves in tea plants. The positioning of *q10.3* in the promoter of *CsTPR* also indicated that CsTPR might function at the transcriptional level, and further exploration is needed to investigate the chromatin accessibility in the promoter of *CsTPR*.

### Genome-wide analysis for accessible chromatin regions of CsTPR according to ATAC-seq

**Figure 2:**
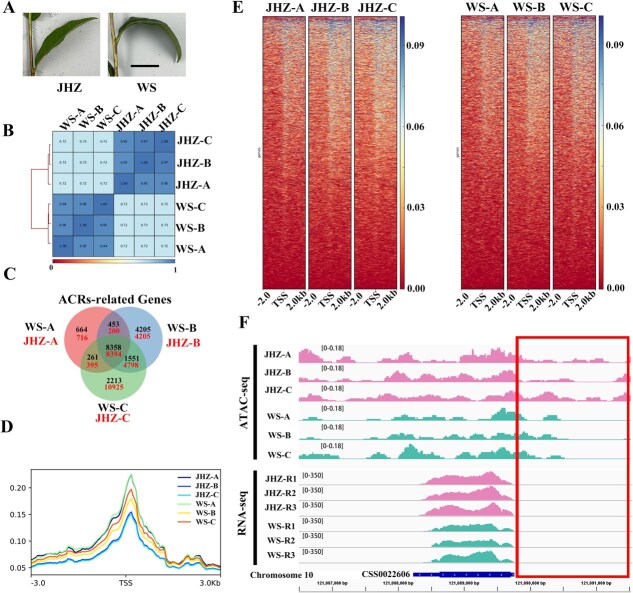
Chromatin accessibility landscapes of droopy leaves and the relationship between chromatin accessibility and gene expression of *CsTPR*. (A) Representative images of leaves from JHZ and WS. Scale bar represents 5 cm. (B) The heatmap and clustering of Spearman correlation of JHZ and WS. Each cultivar has three biological replicates. (C) Venn diagram of accessible chromatin regions (ACRs) related genes in three biological replicates of JHZ and WS. (D) Plots of ATAC-seq signals on both 3-kb sides of the TSS. (E) Average heatmaps of ATAC-seq signals on both 2-kb sides of the TSS. (F) The ATAC-seq and RNA-seq tracks on *CsTPR* (CSS0022606), as shown in IGV view. The box suggested the promoter region of *CsTPR*.

As the higher expression levels of *CsTPR* and the coverage of LD decay extent in coding and promoter region of *CsTPR*, ATAC-seqs with three replicates in tender leaves of ‘JHZ’ (JHZ-A to C) and ‘WS’ (WS-A to C) were conducted to explore the chromatin accessibility of *CsTPR* on a genome-wide scale (Supplemental Fig. S4). Among the 130 accessions studied, JHZ exhibited straight leaves while WS had droopy leaves, both having almost the same length of leaf vein (Fig. 2A). The maximum length of vascular tissue in the leaf vein of WS was shorter than that in JHZ [3], which contributed to the droopy appearance (Fig. 2A). The Spearman correlation heatmap with values suggested a high repeatability in these ATAC-seq results (Fig. 2B). A total of 8394 common genes in the accessible chromatin regions of three replicates were identified for JHZ, while WS had 8358 common genes (Fig. 2C).

The ATAC-seq signals were then determined in the ±3-kb region surrounding transcriptional starting sites (TSS) and the highest signal intensity was primarily located at the TSS (Fig. 2D and E), which was the same with former studies in tea plants [19]. A more detailed analysis of ATAC-seq and RNA-seq focused on the promoter and coding region of *CsTPR* from chromosome 10 (Fig. 2F; Supplemental Fig. S5). The ATAC-seq signals could be detected across both the coding region and promoter region in every sample. The expression levels of *CsTPR* in JHZ were higher than that of WS (Fig. 2F). Additionally, chromatin accessibility within the promoter region of *CsTPR* in JHZ (as the red box indicated) was more open when compared to WS (Fig. 2F). This finding suggests that increased chromatin openness in the promoter region positively influences the expression of *CsTPR*. Altogether, these results confirmed the differential expression pattern of *CsTPR* in droopy leaves, and the roles of CsTPR ought to be revealed with more experiments in tea plants.

### *CsTPR* repressed droopiness of tea leaves at transcriptional level

**Figure 3:**
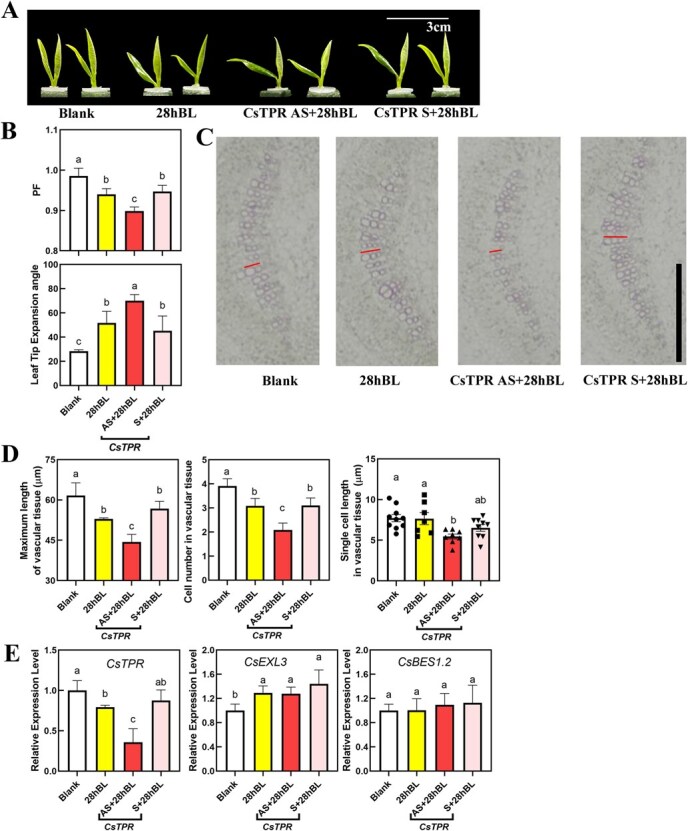
Morphological characterization, physiological characterization, and related gene expression levels of *CsTPR*-silencing tea plant. (A) Representative pictures of *CsTPR* -silencing tea plant. Blank, blank control. 28hBL, control group. *CsTPR* AS, experimental group for silencing *CsTPR* expression. *CsTPR* S, negative control. Scale bar represents 3 cm. (B) PF (the ratio of PDD/FL) and leaf tip expression angle for *CsTPR*-silencing tea plant. (C) Lignin staining on cross-sections of the middle part in leaf blades midrib of *CsTPR* -silencing tea plant with phloroglucinol-HCl. Scale bar represents 100 μm. Lines indicated the maximum length of vascular tissue. (D) Maximum length of vascular tissue, cell number in vascular tissue, and single-cell length in vascular tissue for *CsTPR*-silencing tea plant. (E) Relative expression level of *CsTPR*, *CsEXL3*, and *CsBES1.2* in *CsTPR* -silencing tea plant. For (B) and (D), values are means ± SD of six biological replicates. For (E), values are means ± SD of four biological replicates. Statistical analysis was performed with analysis of variance (ANOVA). Bars with different letters indicate significant difference (*P* < 0.05).

To confirm the role of *CsTPR* in regulating leaf droopiness, we silenced *CsTPR* expressing in the bud of tea plants through using antisense oligonucleotides (AsODN). Exogenous BR treatment (28hBL, 28-homobrassinolide) was applied to accelerate droopiness during the short-term AsODN experiment as previous studies described [3]. The 28nBL treatment promoted leaf droopiness and increased leaf tip expansion angle (Fig. 3A and B), which aligns with prior reports [3].

The 28hBL-induced droopiness and the size of leaf tip expansion angle were significantly enhanced in *CsTPR*-silencing tea plant with AsODN (CsTPR AS group) (Fig. 3A and B). The greater droopiness observed in the CsTPR AS group was in accordance with the leaf blade curling and a downregulation of *CsTPR* expression in the WS group (Fig. 2). Phlorophenol staining was performed to figure out the cellular-level variations in leaf profiles, specifically in the midrib cross-sections. In the 28hBL-treated tea plants, the maximum length of the vascular tissue was shorter than that observed in the blank group and CsTPR S + 28hBL, which served as a negative control (Fig. 3C and D). The cell number in vascular tissue was also less than that observed in the blank group and CsTPR S + 28hBL (Fig. 3C and D). The single-cell length in vascular tissue was also less than that observed in the blank group and CsTPR S + 28hBL, (Fig. 3C and D), but the single-cell length in 28hBL-treated group had no difference with the blank group. *CsTPR*-silencing exacerbated 28hBL-induced droopiness and the maximum length of vascular tissue in the *CsTPR*-silencing group was decreased by ~25% when compared to the blank group and was also much shorter than the other two groups (Fig. 3D).

*CsEXL3* and *CsBES1.2* have been reported to play roles in 28hBL-induced leaf droopiness in tea plants [3]. To investigate the relationship between these genes and *CsTPR*, the expression of droopiness-related genes in tea plants was also assessed (Fig. 3E). The expression level of *CsTPR* was decreased after 28hBL treatment when compared to the blank group. Additionally, the expression level of *CsTPR* was significantly downregulated in the *CsTPR*-silencing group when compared to both the control (28hBL) and negative control (CsTPR S + 28hBL). In the negative control, the expression level of *CsTPR* was kept at the same level as in the control group. These results confirmed the effectiveness of the oligonucleotide silencing on *CsTPR*. The expression level of *CsEXL3* was uplifted under 28hBL treatment but was unaffected by *CsTPR*-silencing in tea plants (Fig. 3E), indicating the repression of CsTPR on droopiness might be independent of CsEXL3. Furthermore, the expression level of *CsBES1.2* was not changed in either the 28hBL treatment group or *CsTPR*-silencing (Fig. 3E). This observation was consistent with the expression level of *CsBES1.2* in *CsEXL3*-silenced tea plants (Supplemental Fig. S8) and indicated that *CsTPR* might not be an upstream factor for CsBES1.2.

### A critical base mutation in the promoter of *CsTPR* might be associated with the expression level of droopiness-related gene, *CsTPR*

To examine the potential structure mutations that caused the different expression levels of *CsTPR*, whole-genome sequencing (WGS) was conducted on two accessions: JHZ, which has straight leaves, and WS, which has droopy leaves. The average sequencing depth across the 15 chromosomes in JHZ and WS were 10.18× and 9.77×, respectively (Supplemental Figs S6 and S7). The length of mapped reads to reference genome and mapping rate in JHZ were 232003167 bp and 97.45%, respectively. For WS, the mapped reads totaled 213819123 bp, with a mapping rate of 96.54%. The coverage rate of at least 1× of JHZ and WS were 90.26% and 88.35%, respectively. These results of WGS met the standard for the consequent analysis.

**Figure 4:**
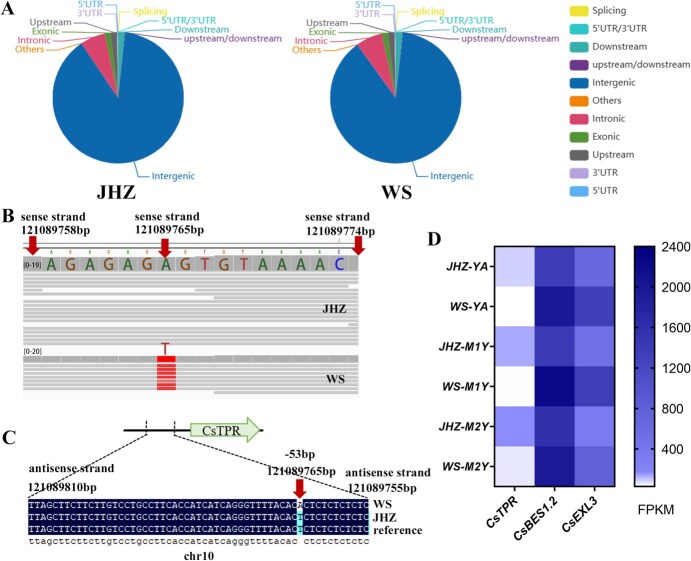
The promoter structure and expression level of *CsTPR*. (A) The number of SNPs in different regions of JHZ and WS. (B) The structure of the CsTPR promoter region (chromosome 10, 121089758–121089774 bp) in the sense stand of WS and JHZ according to WGS. (C) The structure of the CsTPR promoter region (chromosome 10, 121089810–121089755 bp) in antisense stand of WS, JHZ, and SCZ as the results of chromosome walking. The mutant site between JHZ and WS was located at −53 bp (chromosome 10, 121089765 bp) to the initiation site. (D) The expression level of *CsTPR*, *CsEXL3*, and *CsBES1.2* in three types of tissue of JHZ and WS according to RNA-seq. YA, the apical bud and first leaf; M1Y, first mature leaves; M2Y, second mature leaves.

After calling SNP sets via SAMtools, the number of SNPs in different genome regions of JHZ and WS was organized and shown in Fig. 4. The distribution of SNPs across different regions was nearly identical between JHZ and WS (Fig. 4A). To explore the reason causing the difference between the expression levels of *CsTPR* in JHZ and WS, the SNPs located in the promoter region were subsequently examined, particularly within the area of LD decay ranging from 121089783/121089905 bp (blue asterisk indicated in Fig. 1C) to 121089775 bp (initiation of CsTPR). A single-base mutation (A–T on the sense strand) was detected in 121089765 bp of chromosome 10, positioned at −53 bp upstream of the CsTPR initiation site (Fig. 4B). This mutation is also located in the conserved binding site of BZR/BES family genes according to the search result of the upstream region (−2000 bp) for *CsTPR* in JASPAR (http://jaspar.genereg.net/) (Fig. 4B). This conserved binding site is the only one predicted binding site by using JASPAR. To verify the authenticity of this base mutant, chromosome walking was performed in the candidate promoter region for both JHZ and WS. The results of chromosome walking cloning confirmed that this single-base mutation was indeed present in 121089765 bp of chromosome 10 (Fig. 4C; Supplemental Fig. S9). The RNA-seq results showed that the expression level of *CsTPR* was decreased and the expression level of *CsBES1.2* was increased in both three tissues of WS than that of JHZ, which indicates a negative correlation between *CsTPR* and *CsBES1.2* at expressing level (Fig. 4D). Additionally, this negative relationship between *CsTPR* and *CsBES1.2* (Fig. 4D) contrasts with the positive relationship observed between *CsBES1.2* and *CsEXL3* [3], another target gene of CsBES1.2 that regulates droopy leaves in tea plant.

**Figure 5:**
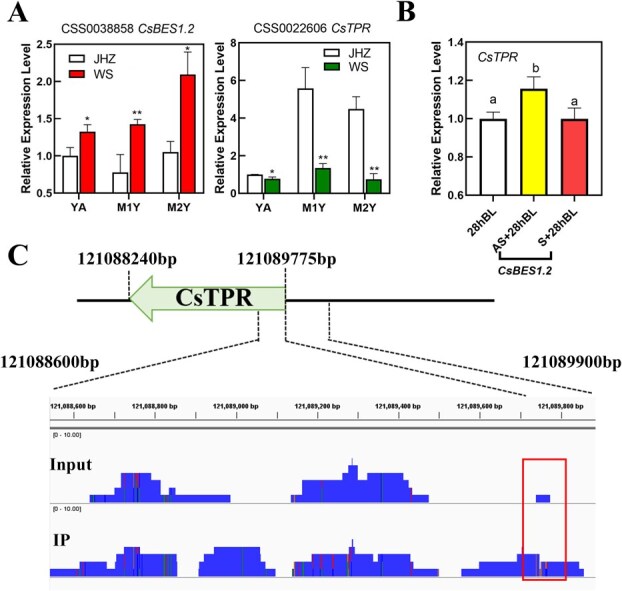
The relationship between the expression levels of *CsTPR* and *CsBES1.2*. (A) Relative expression levels of *CsTPR* and *CsBES1.2* in three types of tissue in JHZ and WS according to real-time qPCR. YA, the apical bud and first leaf; M1Y, first mature leaves; M2Y, second mature leaves. Values are means ± SD of three biological replicates. Statistical analysis was performed with ANOVA. ^**^P < 0.01. ^*^P < 0.05. (B) Relative expression levels of *CsTPR* in *CsBES1.2*-silencing tea plant. 28hBL, control group. CsBES1.2 AS, the experimental group for silencing *CsBES1.2* expression. CsBES1.2 S, negative control. Values are means ± SD of four biological replicates. Statistical analysis was performed with ANOVA. Bars with different letters indicate a significant difference (*P* < 0.05). (C) A screenshot of the ChIP-seq profile of CsBES1.2 at the promoter and coding region of *CsTPR*. The box indicates the binding site of CsBES1.2 on the promoter of *CsTPR*.

Quantitative real-time polymerase chain reaction (PCR) experiments were performed for *CsTPR* and *CsBES1.2* (Fig. 5A) and the results of qPCR for *CsTPR* and *CsBES1.2* were in accordance with the results of RNA-seq. The expression level of CsTPR was upregulated in the *CsBES1.2*-silencing tea plant when compared to the control and negative control (Fig. 5B), which suggested CsBES1.2 might negatively regulate the expression of *CsTPR*.

Considering these results, CsBES1.2 might repress the expression level of *CsTPR* by binding the site that contains the detected single-base mutation. This single-base mutation in the promoter of CsTPR might account for the expressing gap between JHZ with straight leaves and WS with droopy leaves. To verify this possibility, chromatin immunoprecipitation sequencing (ChIP-seq) of CsBES1.2 was accomplished in tender leaves of JHZ by polyclonal antibodies of CsBES1.2 (Fig. 5C). The results of ChIP-seq demonstrated significant enrichment of CsBES1.2 on the 121089780–121089900 bp of the *CsTPR* promoter region in chromosome 10. Interestingly, the below single-base mutation at 121089765 bp happened to be within this enriched region. This coincidence indicates that this single-base mutation might disrupt the binding of CsBES1.2 on the promoter of *CsTPR* and then impact the expression level of *CsTPR*.

Overall, CsBES1.2 might repress the expression of *CsTPR* by binding the 121089600- to 121089800-bp region of the *CsTPR* promoter in chromosome 10. The single-base mutant within this binding region might interfere with binding activity and therefore disturb the expression of *CsTPR*.

### CsBES1.2 inhibited the expression of *CsTPR* via binding the promoter of *CsTPR* and a single-base mutant enhanced this binding capacity

**Figure 6:**
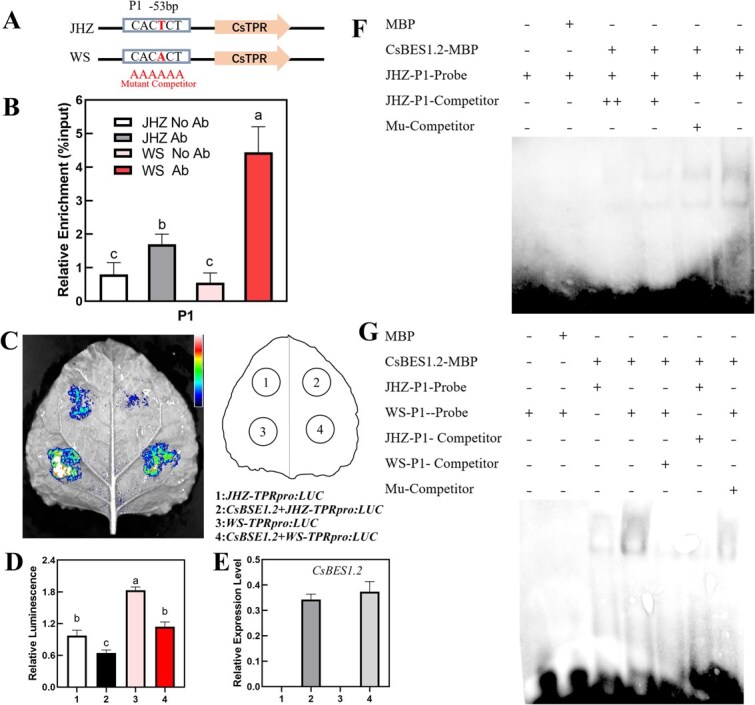
The transcription regulation of CsBES1.2 on the expression of CsTPR through binding two types of promoter. (A) Schematic diagram of *CsTPR* promoters in JHZ and WS, indicating the amplicons used for ChIP-qPCR. Square frame indicated the binding site, P1. The difference of SNP between JHZ and WS was indicated in the square frame. The mutant competitor (Mu-Competitor) sequence was shown below the position of P1. (B) ChIP-qPCR assays of CsBES1.2 on the selected promoter region of *CsTPR* in JHZ and WS. This selected promoter region included P1. The result of CsGAPDH served as control. The relative enrichment for the ChIP signal was displayed as the percentage of total input DNA. Values are means ± SD of three biological replicates. Statistical analysis was performed with ANOVA. Bars with different letters indicate a significant difference (*P* < 0.05). (C) Transient expression assays shows that CsBES1.2 represses *CsTPR* expression. Representative images of *N. benthamiana* leaves were taken at 48 h after infiltration. The right panel indicates the infiltrated constructs. (D and E) Luminescence intensity (D) and *CsBES1.2* expression level (E) under different treatments as indicated in (C). Values are means ± SD of six biological replicates. Different letters indicate significant differences among groups for each locus (one-way ANOVA with Tukey’s test, *P* < 0.05). (F) DNA EMSA showing the binding of CsBES1.2-MBP to P1 of JHZ *CsTPR* promoter *in vitro*. (G) EMSA showing that the binding of CsBES1.2-MBP to P1 of WS *CsTPR* promoter and the binding activity comparison of CsBES1.2-MBP between WS and JHZ on P1 of *CsTPR*. Biotin-labeled probes were incubated with CsBES1.2-MBP and the free and bound DNAs were separated on an acrylamide gel. An equal amount of CsBES1.2-MBP was added in each lane.

To affirm the possibility that the downregulation of CsBES1.2 and the effect of identified single-base mutation inside the binding region on the expression of *CsTPR*, we conducted further experiments, including ChIP-seq, transient expression assays, and electrophoretic mobility shift assays (EMSA). The predicted binding regions of CsBES1.2 on the promoter of *CsTPR* in JHZ and WS were designated as P1 and illustrated in Fig. 6A. The identified single-base mutation inside P1, located at −53 bp of the *CsTPR* promoter, was T in the JHZ-type promoter and A in the WS-type promoter, respectively (Fig. 6A). Further results of chromatin immunoprecipitation quantitative polymerase chain reaction (ChIP-qPCR) showed that CsBES1.2 bound on P1 of the *CsTPR* promoter in both JHZ and WS. The binding enrichment of CsBES1.2 on P1 was much stronger in WS (~8.07-fold) than that of JHZ (~2.12-fold) (Fig. 6B).

To independently verify the effect of CsBES1.2 on the transcriptional function of *CsTPR* by using the transient expression assay, we generated a *JHZ-TPRpro:LUC* reporter, in which luciferase (LUC) was fused with the JHZ-type *CsTPR* promoter, and a *WS-TPRpro:LUC* reporter, in which LUC was fused with the WS-type *CsTPR* promoter following described single-base mutant. When the *TPRpro:LUC* reporter was co-expressed with *CsBES1.2*, luminescence intensity was significantly inhibited (Fig. 6C–E), which suggested that CsBES1.2 transcriptional depressed the expression of *CsTPR* in both JHZ and WS. The luminescence intensity of *WS-TPRpro:LUC* was stronger than that of *JHZ-TPRpro:LUC*, which indicated a more powerful promoter activity of *CsTPR* in WS. More importantly, the restraining degree for *CsBES1.2* construct on *WS-TPRpro:LUC* (~0.71-fold) was slightly stronger than that on *JHZ-TPRpro:LUC* (~0.63-fold), which suggested that the inhibition function of CsBES1.2 on *CsTPR* expressing in WS was more powerful than that in JHZ. This regulating gap was consistent with the difference in binding capacity shown in ChIP-qPCR *in vivo* (Fig. 6B).

EMSA was then performed to confirm the binding ability of CsBES1.2 on the promoter of *CsTPR* and the capacity difference between JHZ- and WS-type promoter of *CsTPR*, which contains P1. CsBES1.2-MBP bound to the P1 of the *CsTPR* promoter (Fig. 6F), which was in accordance with the results of ChIP-seq and ChIP-qPCR (Figs 5C and 6B). This binding on P1 could be outcompeted by an unlabeled probe instead of the probe without P1. Based on the bands’ intensity, the binding of CsBES1.2 on the WS-type P1 probe was significantly stronger than that of the JHZ-type P1 probe (Fig. 6G), reinforcing the findings from ChIP-qPCR and transient expressing assay. The binding capacity gap might be a key factor that leads to the regulation discrepancy of CsBES1.2 on the expression of *CsTPR*.

In summary, CsBES1.2 transcriptionally suppressed the expression of *CsTPR* through binding P1 within its promoter. The single-base mutation at −53 bp inside P1 enhanced the binding ability of CsBES1.2, thereby strengthening its suppression effect on the expression of *CsTPR*.

## Discussion

BES1, which serves as the core component of BR signaling, orchestrates a regulatory network with both the inhibition and promotion of specific traits. AtBES1 transcriptionally regulated target genes in both directly positive and negative ways in *Arabidopisis* [20]. In this paper, a BES1 homologous gene, CsBES1.2, has been proven to directly downregulate the expression level of a target gene, *CsTPR*, to module leaf droopiness in tea plants (Figs 3 and 6). This negative regulation contrasts with the positive regulation of CsBES1.2 on *CsEXL3* in the tea plant [3]. From another perspective, CsBES1.2 regulated droopiness by simultaneously repressing and inducing gene expression. This dual regulation appears utterly distant from the reported behavior of BES1, which can positively regulate one trait while negatively regulating another [21,22]. However, these opposite regulations of BES1 on the same trait were in accordance with the complex regulation of AtBZR1, a member of the BES1/BZR1 family genes, on cell elongation in *Arabidopsis*. In detail, AtBZR1 repressed the inhibition of BZS1 and IBH1 on cell elongation through binding the promoter of *BZS1* and *IBH1* [23]. Meanwhile, AtBZR1 directly promoted the expression of positive regulatory on cell elongation, such as PRE1 and BEE1, via binding of their promoters [23]. AtBZR1 ultimately directly promotes cell elongation with both positive and negative regulation.

The conserve binding motif of BES1 on the promoter of target genes included BRRE-box and E-box in various plant species, such as tomato, rice, and tea plant [22,24,25]. The strengths of binding capacity on these two elements vary across different traits in various crops. In *Arabidopsi*s, the binding capacity of BES1 on the BRRE-box is stronger than that of the E-box [20]. However, in the tea plant, the binding capacity of CsBES1.2 on the BRRE-box is significantly weaker than that of the E-box [3]. SlBES1 repressed the target gene mainly through the E-box rather than the BRRE-box in tomato [22]. This evidence suggested that the strength of binding capacity on the target motif might not be conserved across species. In comparison to the BRRE-box (CGTGT/CG), the criteria for identifying the E-box (CANNTG) are less stringent, suggesting that the E-box is a more prevalent binding element within the promoter of target genes that are related to specific traits in different crops. For instance, the E-box instead of the BRRE-box was figured out in the promoter of droopiness-related gene, *CsTPR*, in this study (Fig. 6). The E-box was reported to be the only binding element type for NtBES1 on promoters of NtMYB27 to modulate flavonoid accumulation in tobacco [26]. Moreover, when focusing on the drought tolerance of maize, the E-box seemed to be the ubiquitous binding element for ZmBES1 [27]. Additionally, the E-box, rather than the BRRE-box, was identified as the binding element for GhBES1 on the promoter of *GhCERP* in cotton [21].

With the increase of transcriptional regulation-related research [28], the role of E-box in deciding regulation direction is constantly changing. Initially, the E-box was regarded as an enhancer element for its location in the promoter of BES1-induced target genes in Arabidopsis [24]. However, the role of E-box in increasing gene expression was totally opposite in various species. For example, BES1/BZR1 family genes repressed the expression level of *FC1* via binding E-box in rice [25]. SlBES1 downregulated the expression of *PMEU1* by binding E-box in tomato fruit [22].

The droopiness usually results in a larger leaf tip expansion angle, also described as blade angle (from leaf tip to node), in tea plants or other plant species. *CsTPR*-silencing tea plant had droopy leaves with larger leaf tip expansion angle (Fig. 3A). *CsEXL3*-silencing tea plant had straight leaves with smaller leaf tip expansion angle, which phenocopied *CsBES1.2*-silencing tea plant [3]. In *Setaria*, *dpy1* with an extremely droopy leaf phenotype has a significantly enlarged leaf tip expansion angle [29]. But, it is uncertain that leaf with the larger leaf tip expansion angles have droopiness trait as other crops revealed. For example, the regulation of blade angle does not seem to have muted the droopiness in LIC-related lines of rice [30]. This independent relevance from the larger leaf tip expansion angles to droopiness still need to be uncovered at more studies with the beginning of blade angles instead of droopiness. In conclusion, our findings fulfilled the regulation network of CsBES1.2 on leaf droopiness in tea plant and the roles of E-box in positive and negative regulation of BR signal components on specific traits.

## Materials and methods

### Tea plants and growth conditions

Collected tea plant (*C. sinensis* (L.) O. Kuntze) accessions from NTGRH were located at Hangzhou, Zhejiang, China. These accessions included cultivars from Jiangsu Province, such as XC5 (‘Xicha 5’) and ‘Xicha 11’ (Supplemental Table S1). After photography and anatomical observation, frozen leaves with liquid nitrogen were saved as samples at a −80°C ultra-low-temperature freezer for consequent analysis.

### Determination of PDD/FL ratio and leaf tip expansion angle

PDD (proximal–distal distance)/FL (blade full length) ratio (PF) was set as index for leaf droopiness. PF and leaf tip expansion angle of tea plant shoots from pictures were obtained by ImageJ and the values for ratio or angle were then calculated. Diagrams of PDD, FL, and leaf tip expansion angle were shown in Supplemental Figs S1 and S2.

### Genome-wide association study

One hundred thirty accessions and detailed methods were applied for GWAS as previously described [3]. ‘Shuchazao’ V2 was designated as the reference genome. PF was used as trait index. A mixed linear model implemented in EMMAX program was applied for GWAS and Bonferroni correction was used to calculate *P*-value thresholds. Then the threshold >8.6 was set as limits to determine significant loci threshold by GEC software. LD plot for candidate genotyping region had been generated by R library LDheatmap.

### Anatomical observation of tea leaves

The midrib cross-section was obtained through scalpel dissection at its median region. Then, the FAA solution was applied to fix the selected sections at 4°C overnight. The treated tissue underwent sequential ethanol dehydration (10%–100% gradient) followed by embedding. Ten micrometer sections on slides were coated with polylysine. Subsequently, tissue sections were subjected to phloroglucinol-HCl staining (1% w/v phloroglucinol in 12% HCl) for 5 min, followed by immediate microscopic observation using a Nikon light microscope (Tokyo, Japan). The lignin relative content was calculated based on the shade of phloroglucinol-HCl-stained cells in the software ImageJ.

### Vector construction

Vectors were constructed and generated as previous research showed [3]. To verify the authenticity of CsTPR and CsBES1.2, their complete coding sequences were amplified using KOD plus Neo polymerase in a PCR reaction and subsequently cloned into the pEASY-Blunt Zero Cloning Vector. The former recombinant vector, pMAL-c5x-CsBES1.2, was used [3]. Primers were listed in the Supplemental Table S3.

### Whole-genome sequencing for tea plant

WGS was performed by Novogene (Beijing, China) and ‘Shuchazao’ V2 genome was set as reference genome. Briefly, a total amount of 0.2 μg DNA was extracted from tender leaves of JHZ and WS for DNA library preparation. The sequencing library was generated from sonicated 350-bp DNA fragments according to NEB Next® Ultra™ DNA Library Prep Kit for Illumina (NEB, USA). After clustering and sequencing on the Illumina platform, reads were mapped to reference sequences and annotated through BWA and ANNOVAR software, respectively. Samtools software was applied to obtain SNP. Finally, a total of 36.77 and 34.19 G raw data were obtained for JHZ and WS, respectively. Other software and related parameters had been listed in Supplemental Table S4.

### Chromosome walking for promoter cloning

After obtaining the true CDS sequence of CsTPR, the promoter regions of CsTPR in JHZ and WS were amplified following the KX genome walking kit (Zoman Biotechnology, Beijing, China). Primer SP1 5′ CTCTTTCTCTTCGTCCGAACCAGCT 3′, SP2 5′ TAGGAGAGAGAAAGGGAAAGGGTTT 3′ and SP3 5’ GGTGATGAAGATGCTCTGATTGAGA 3′ were designed according to CDS sequence and used for genome walking cloning.

### Quantitative real-time PCR

EASYspin Plus Complex Plant RNA Kit (Aidlab, Beijing, China) was used to extract RNA from 0.2 g selected liquid nitrogen-frozen tea leaves. cDNA was then synthesized by reverse-transcribing of PrimeScript RT reagent (Takara, Kusatsu, Japan) on extracted RNA. These cDNA and SYBR Green (Roche, Basel, Switzerland) were utilized for quantitative real-time PCR in Lightcycler 480 II (Roche, Basel, Switzerland). qPCR was performed with 10 μl of mixture, which contained 1 μl of cDNA, 0.4 μl of each 5 μM forward and reverse primer, 3.2 μl of double-distilled water, and 5 μl of SYBR Green. PCR amplification was performed using cycling conditions of 95°C for 10 s, followed by 45 cycles of 94°C for 10 s, 58°C for 15 s, and 72°C for 12 s.

### RNA-seq

The apical bud and 1st leaf (YA), first mature leaves (from top to bottom, M1Y), and second mature leaves (M2Y) of JHZ49 or WS231 were collected from NTGRH for RNA extraction and the Illumina MiSeq library was prepared following the manufacturer’s protocol (Illumina, San Diego, CA, USA) [ 31] and subsequent sequencing was performed on the Illumina NovaSeq 6000 platform via using a paired-end 150-bp configuration. Software for further analysis was described as previous research shown [32]. Three replicates for each kind of tissue in each cultivar were set.

### Chemical induction and gene silencing in tea plants

The apical bud and first leaf of JHZ were treated with 5 μM 28-hBL (28-homobrassinolide) or AsODN as former study described [3]. A total of 20 μM AsODN of CsTPR and CsBES1.2 were synthesized and applied for 3 days. The blank group and control group were both set following the previous description [3]. Six independent biological replicates were performed for each experimental group. The used sequence for AsODN and random nonsense ODN were listed in the Supplemental Table S3.

### ChIP quantitative PCR and sequencing

ChIP-qPCR and ChIP-seq in JHZ or WS were carried out based on CUT&RUN Assay Kit (Vazyme, Nanjing, China). Peptide chains of CsBES1.2 (RGSKRKADWESFSNGS) were synthesized and used as an antigen to generate polyclonal antibodies in rabbits through ABclonal Biotechnology (Wuhan, China). The immunoprecipitation test had been done to confirm validation of polyclonal antibodies for CsBES1.2, as shown in Supplemental Fig. S10. Three biological replicates (each containing two technical replicates) were used for ChIP-qPCR. CsGAPDH acted as a control gene to calculate fold enrichment. Primers for ChIP-seq were listed in the Supplemental Table S3. ChIP-seq was widely applied in many crops [33,34]. ChIP-seq in this study was generated and analyzed according to the previous study [3]. Detailed methods were described in supplemental materials. Related parameters had been listed in Supplemental Table S4.

### ATAC-seq

Sample preparation and library for ATAC-seq were conducted as previously described [35] with modification. In brief, tender leaves were ground with 1 ml of 1 × HB buffer and then centrifuged at 4°C 500 × g for 5 min. Sediment was gradient-centrifuged for 10 min to get nucleus suspension and further counted with LUNA-FL. A total of 50 000 nuclei were selected and mixed with 1 ml washing buffer. Following centrifugation at 2100 rpm for 5 min, the nucleus precipitate was resuspended in the Tn5 transposase reaction mix buffer. After incubation of transposition reaction at 37°C for 30 min, PCR was performed with equimolar Adapter 1 and Adapter 2 by ATAC-seq Novogene Kit. PCR-generated libraries were purified with the AMPure beads and the library quality was evaluated by Qubit fluorometric quantification. Software and related parameters had been listed in Supplemental Table S4.

### Electrophoretic mobility shift assay

EMSA was conducted following the previous study described [3]. CsBES1.2-MBP protein was purified from *Transetta* (DE3) cells that were expressing pMAL-c5x-CsBES1.2. Oligonucleotide probes for CsTPR promoters were labeled with biotin at 5’ end. Detailed information was mentioned in Supplemental Table S3.

### Transient expression assay

A transient expression assay was conducted in tobacco (*Nicotiana benthamiana*) leaves following the previously established methodology [22]. CsTPR promoter was cloned upstream of the LUC reporter gene into vector pGWB35 using Gateway cloning kit (Thermo, Boston, USA) to generate the *CsTPRpro:LUC*. Agrobacterium strain GV3101 was transformed with the effector construct *CsBES1.2-1300* and the reporter construct *CsTPRpro:LUC*, supplemented with the P19 silencing suppressor and pSoup helper vector. LUC expression image and LUC luminescence intensity were captured in Tanon 5200 Multi (Tanon, Shanghai, China). Five independent testing points for each sample were performed.

## Supplementary Material

Web_Material_uhaf098

## Data Availability

The genomic and sequencing data associated with this study are available in public repositories as follows: RNA-seq and ChIP-seq data are accessible under NCBI accession PRJNA1075292, while WGS and ATAC-seq data can be retrieved using accession numbers PRJNA1206779 and PRJNA1207240, respectively. The gene sequences of CsTPR (CSS0022606) and CsBES1.2 (CSS0038858) are available in the tea genome database. Additionally, whole-genome sequencing data for 130 tea accessions used in GWAS analysis are publicly accessible through TeaGVD (http://www.teaplant.top/teagvd) and listed in Supplemental Table S1.
