# Supplementary material for: A single-base mutation in promoter of CsTPR enhances the negative regulation on mechanical-related leaf drooping in tea plants
Source: Hortic Res. 2025 Mar 25;12(7):uhaf098. doi: 10.1093/hr/uhaf098 (PMC12087448; doi:10.1093/hr/uhaf098)
Supplement: Web_Material_uhaf098 [file web_material_uhaf098.zip › 250317 supplemental text and figures S1-10 CsTPR .docx]

Detailed Methods for ChIP-seq

In brief, pair-end sequencing of sample was performed on Illumina platform (Illumina, CA, USA) after library construction via Novogene Corporation (Beijing, China). Filtered raw sequencing reads were mapping to tea plant genome (‘Shuchazao’ V2) by BWA mem (v 0.7.12). MACS2 (version 2.1.0) peak calling software was used to estimate fragment size and then detect peak for IP enrichment regions. Finally, peak associated genomic characteristics were annotated by using the R package ChIPseeker and mapped reads visualization were performed by using IGV software.


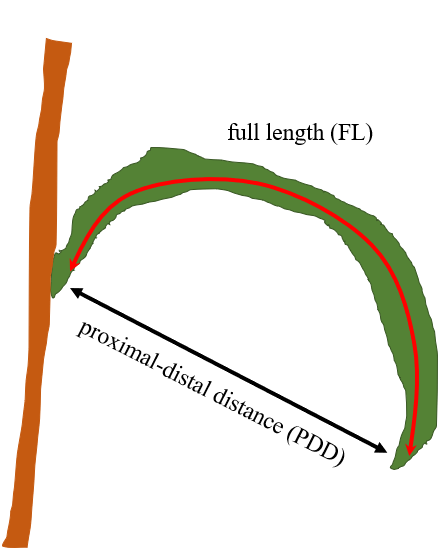
Figure S1. Diagram illustrating the proximal-distal distance (PDD) and full length (FL) of a leaf blade. The ratio (PF) of proximal-distal distance (PDD) to full length (FL) was used to measure curling degree of the leaf blade.


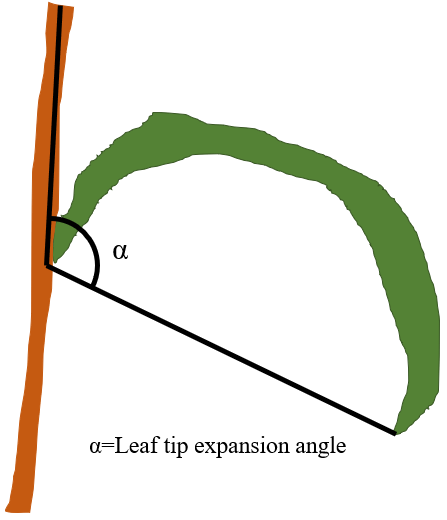


Figure S2. Diagram illustrating the leaf tip expansion angle (α).


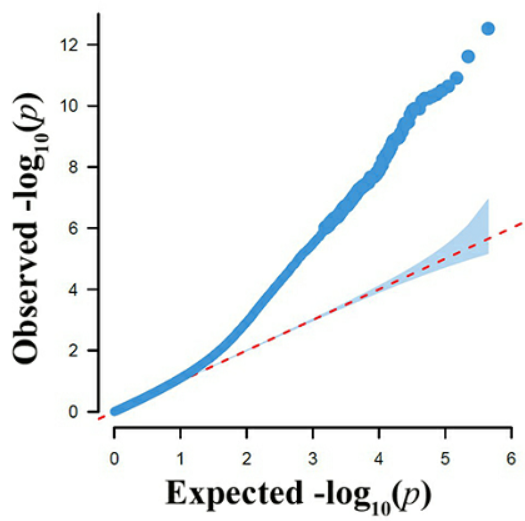


Figure S3. The correlation analysis Q-Q plot according to genome-wide association study for PF.


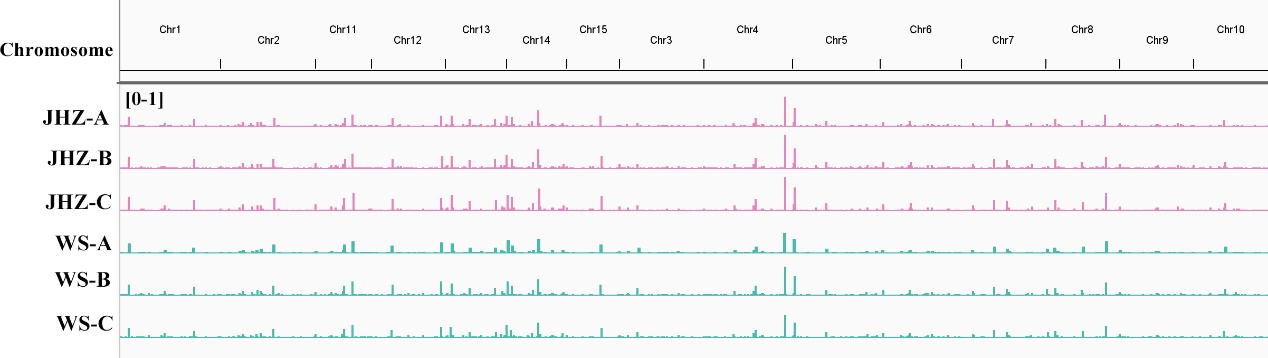


Figure S4. ATAC-seq panorama for all chromosomes in JHZ and WS. JHZ-A, JHZ-B, and JHZ-C were three replicates for JHZ. WS-A, WS-B, and WS-C were three replicates for WS.


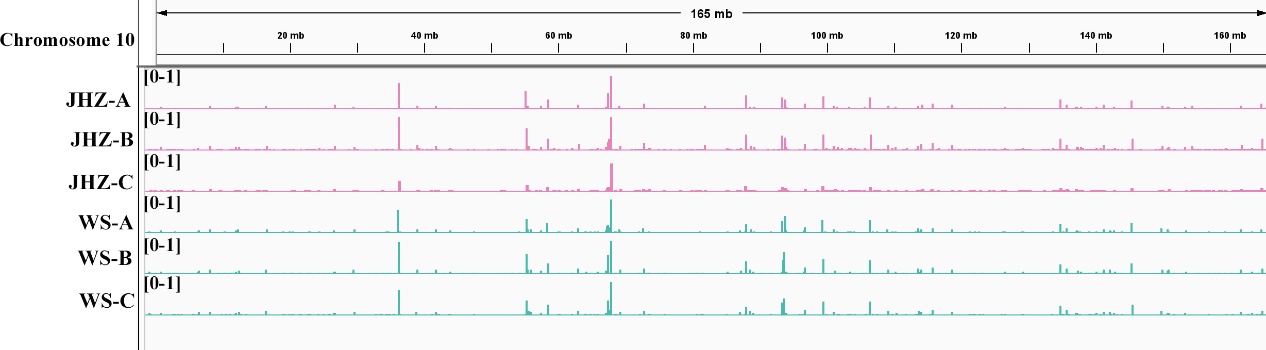


Figure S5. ATAC-seq panorama for chromosome 10 in JHZ and WS. JHZ-A, JHZ-B, and JHZ-C were three replicates for JHZ. WS-A, WS-B, and WS-C were three replicates for WS


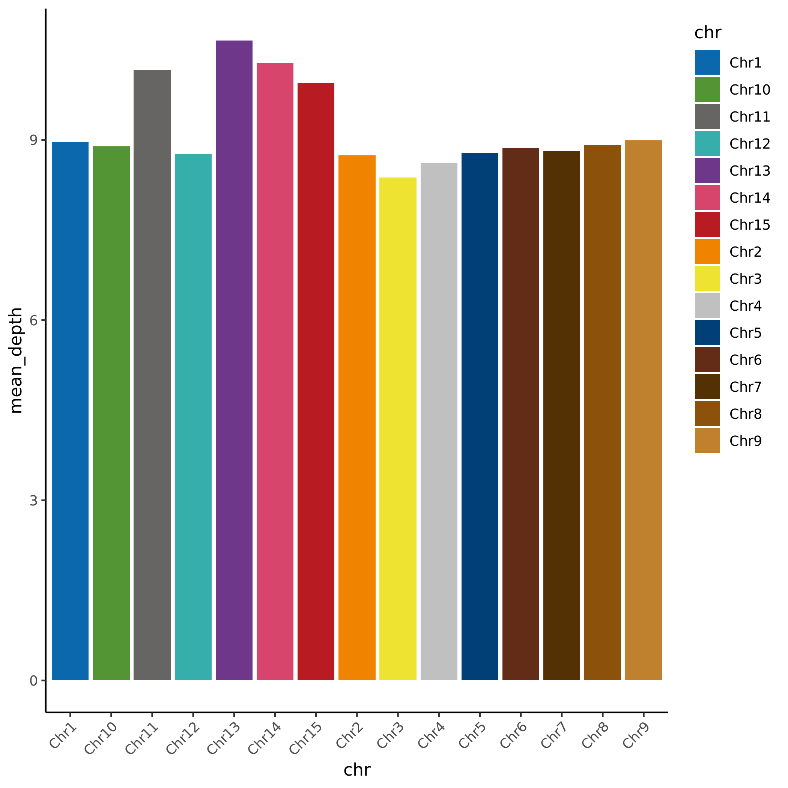


Figure S6. The sequence depth of each chromosome in JHZ


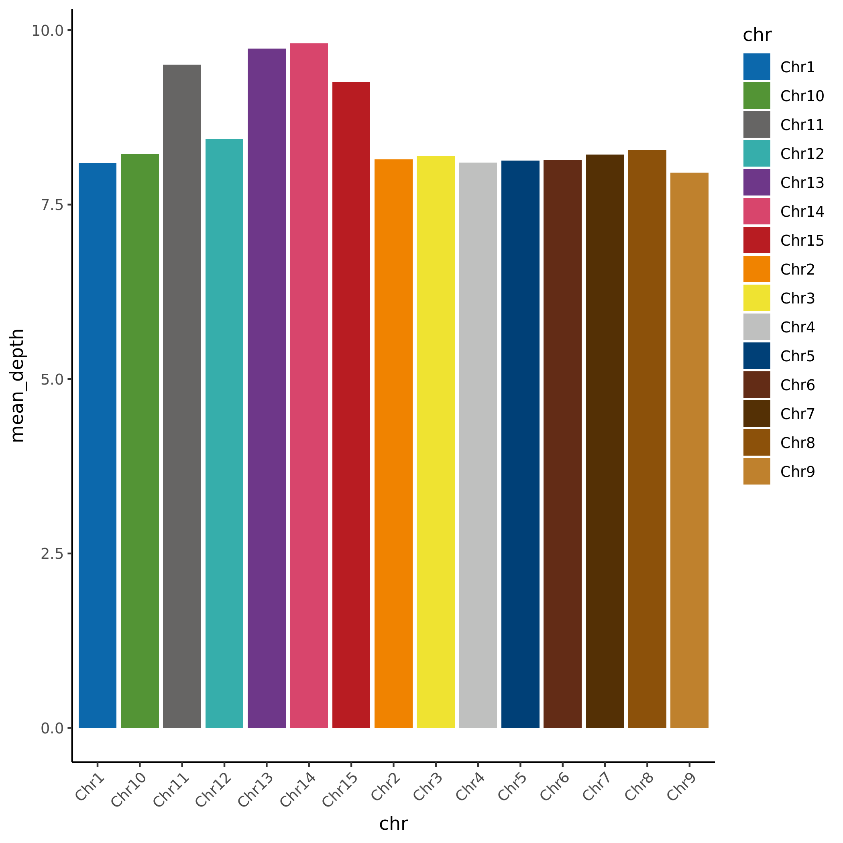


Figure S7 The sequence depth of each chromosome in WS


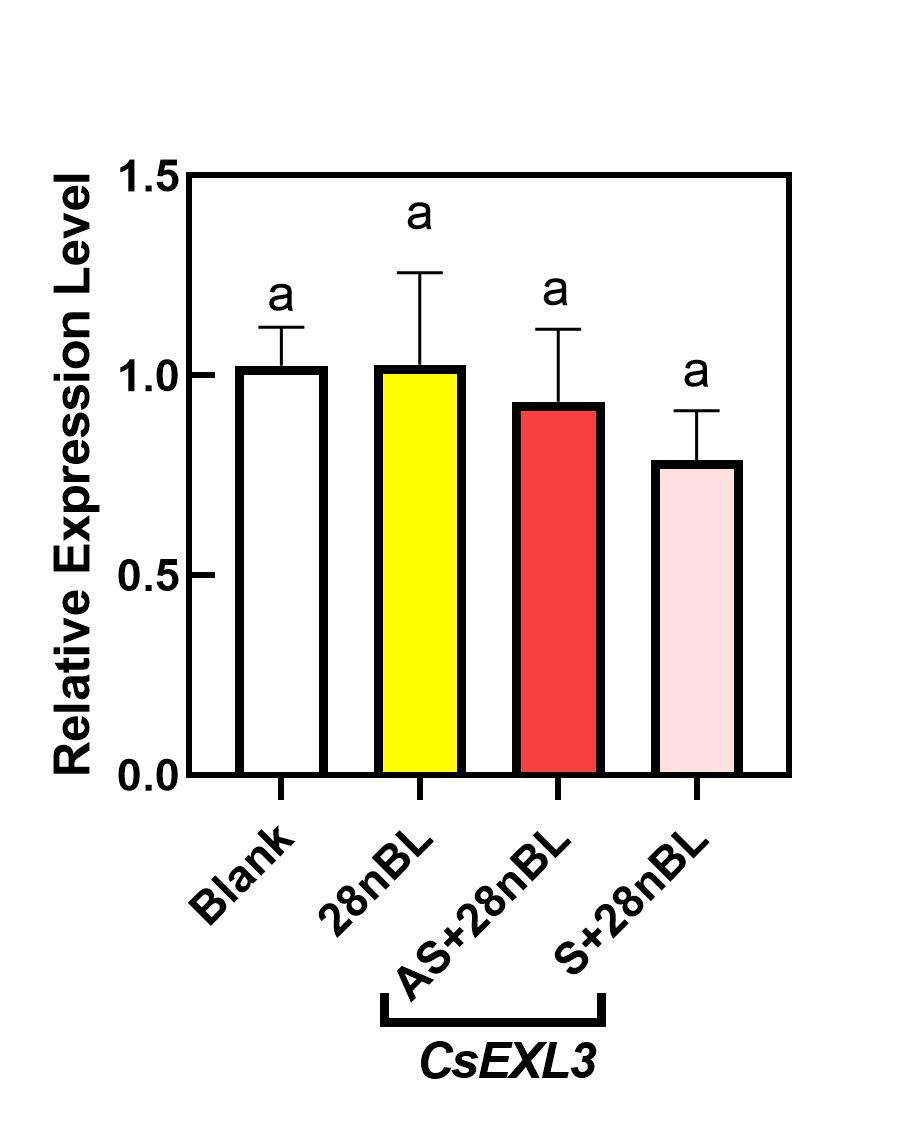


Figure S8 The expression levels of *CsBES1.2* in *CsEXL3*-silencing tea plants.


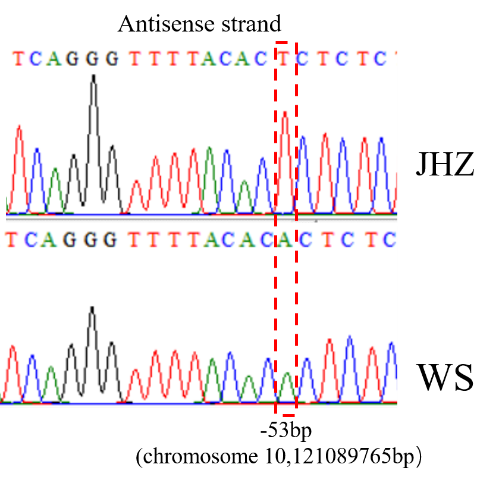


Figure S9 The sequencing peak map for the promoter of *CsTPR* in JHZ and WS. The mutation location at -53bp was indicated with red square frame.


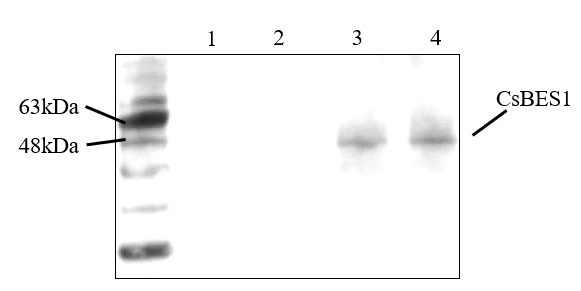


Figure S10. IP test for the self-prepared polyclonal antibodies of CsBES1. Channel 1 and 2, blank control without plant material. Channel 3, IP test for proteins in JHZ. Channel 4, IP test for proteins in WS.
